# Supplementary material for: Country of birth as a potential determinant of inadequate antenatal care use among women giving birth in Brussels. A cross-sectional study
Source: PLoS One. 2022 Apr 15;17(4):e0267098. doi: 10.1371/journal.pone.0267098 (PMC9012396; doi:10.1371/journal.pone.0267098)
Supplement: S1 File — French version. (PDF) [file pone.0267098.s003.pdf]

Migrant Friendly Maternity Care Questionnaire- version adapté

Introduction

Comme expliqué dans le document d'information que vous avez reçu, je travaille pour une équipe de recherche universitaire qui vise à comprendre les soins donnés aux femmes enceintes. Les questions portent sur votre expérience avec les soins de santé pendant la grossesse, l'accouchement, et après la naissance de votre bébé(s). Une partie de ce questionnaire porte sur votre santé et sur votre situation sociale et familiale. Veuillez, s'il vous plaît, m'interrompre si vous avez des questions, ou vous voulez que je répète une question. Toutes les informations que vous partagez restent confidentielles. Vous avez le droit d'arrêter de participer à cette étude à n'importe quel moment. De plus vous avez le droit de ne pas répondre aux questions qui vous mettent mal à l'aise.
Avez-vous des questions -avant de commencer?
Bien, on commence!

**Nº identification:**		**Code d'intervieweur:**	
**Nationalité actuelle:**		**Date de l'entrevue:**	___/___/_____
**Hôpital d'accouchement:**		**Jour après naissance:**	J0, J1, J2, J3, J___
**Mode entretien:**	Hôp□ Tel□ Dom□	**Langue entretien:**	
**Heure début:**		**Interprète:**	Oui □ Non □
**Heure fin:**			

Pour commencer, voici quelques questions d'information générale à propos de vous

1. Quel est votre pays de naissance?

- ☐ Belgique
- ☐ Autre : ______________________

2. Quelle nationalité aviez-vous à votre naissance?

- ☐ Belge
- ☐ Autre :___________________

3. Combien de temps avez-vous vécu en Belgique?

__ __ (années) ___ __ (mois) __ __ (semaines)

(le nombre TOTAL durant lequel la personne a vécu en Belgique)

4. Quel âge avez-vous?

__ __ ans

5. Quel est votre niveau de connaissance du Français ?
(Lisez à voix haute et cochez une option dans chaque ligne. Souvenez-vous de la réponse pour « oral »)

	Couramment	*Bon*	*Avec difficulté*	*Pas du tout*
Oral (parlé et compris)	☐	☐	☐	☐
Lu et écrit	☐	☐	☐	☐

6. Quel est votre niveau de connaissance du Néerlandais ?
(Lisez à voix haute et cochez une option dans chaque ligne. Souvenez-vous de la réponse pour « oral »)

	Couramment	*Bon*	*Avec difficulté*	*Pas du tout*
Oral (parlé et compris)	☐	☐	☐	☐
Lu et écrit	☐	☐	☐	☐

Les questions suivantes concernant votre récente GROSSESSE

7. Durant cette grossesse, avez-vous consulté un médecin, une sage-femme ou un autre professionnel de la santé?

☐ Oui (**dans quels pays:** ☐ Belgique ☐ ailleurs)
☐ Non (**pourquoi pas:** ______________________________) ***(→ Passez à la Q16)***

8. Est-ce que votre grossesse a principalement été suivi par un gynéco, une sage-femme ou tous les deux ?

☐ Gynéco/ obstétricien
☐ Sage-femme
☐ Les deux
☐ Autre (**précisez**:___________________)
☐ S/O

9. A combien de semaines de grossesse avez-vous consulté un gynéco ou une sage-femme pour la première fois?
(Une visite qui avait uniquement pour objectif de faire le test de grossesse ne compte pas)

- ☐ Premiers 2 mois (4-9 semaines) ***(→ Passez à la Q11)***
- ☐ 3ème mois (10-13 semaines) ***(→ Passez à la Q11)***
- ☐ 4ème mois (14-18 semaines)
- ☐ 5ème mois (19-23 semaines)
- ☐ 6ème mois (24-28 semaines)
- ☐ Après le 6ème mois (29+ semaines)
- ☐ Ne sait pas ***(→ Passez à la Q11)***
- ☐ S/O n'a pas consulté

---

10. Pourquoi n'avez-vous pas consulté plus tôt ?
(lisez à voix haute et cochez une réponse)

- ☐ Vous n'aviez pas besoin
- ☐ Vous ne saviez pas à qui vous adresser
- ☐ C'était difficile d'avoir un rendez-vous chez un gynéco ou une sage-femme pour des raisons administratives (problèmes avec la mutuelle, l'AMU ou autre)
- ☐ Vous n'avez pas pu avoir un rendez-vous plus tôt par manque de disponibilité du gynéco ou de la sage-femme
- ☐ Vous ne saviez pas que vous étiez enceinte
- ☐ Autre:____________________________

---

11. Avez-vous rencontré des difficultés pour avoir des rendez-vous avec un gynéco ou une sage-femme?

- ☐ Non
- ☐ Oui (**Précisez**:____)
- ☐ Ne sait pas

---

12. Au total, combien de fois avez-vous consulté le gynéco ou la sage-femme pendant cette grossesse, en excluant les rendez-vous ayant pour but unique de faire une échographie?

- ☐ 1-2 fois
- ☐ 3-6 fois
- ☐ 7-9 fois
- ☐ 10 fois
- ☐ Ne sait pas
- ☐ S/O n'a pas consulté

---

13. Auriez-vous voulu avoir plus de rendez-vous avec le gynéco ou la sage-femme pendant cette grossesse ?

- ☐ Oui
- ☐ Non
- ☐ Ne sait pas
- ☐ S/O n'a pas consulté

14. **Est-ce que, pendant votre grossesse, vous avez été suivie par le même gynéco ou la même sage-femme?**

- ☐ Oui, toujours ***(→ Passez à la Q16)***
- ☐ Oui, la plupart du temps ***(→ Passez à la Q16)***
- ☐ Non
- ☐ Ne sait pas
- ☐ S/O n’a pas consulté ***(→ Passez à la Q16)***

15. **Est-ce que vous aviez l’impression qu’il y avait une bonne continuité dans votre suivi; c’est-à-dire que les gynécos ou sages-femmes qui vous suivaient se relayaient bien l’information ou travaillaient bien ensemble?**

- ☐ Oui, globalement
- ☐ Oui, plus ou moins
- ☐ Non
- ☐ Ne sait pas
- ☐ S/O n’a pas consulté

16. **Avez-vous ou votre bébé eu des problèmes de santé pendant cette grossesse?**

- ☐ Oui, *(laissez répondre et cochez toutes les réponses qui s’appliquent. Puis relancer sur les options avec *)*
  - ☐ Anémie *
  - ☐ Hypertension artérielle (précédant la grossesse ou survenue pendant la grossesse)*
  - ☐ Pre-eclampsie*
  - ☐ Diabète de grossesse*
  - ☐ Infection urinaire*
  - ☐ Dépression*
  - ☐ Nausées et vomissements sévères (hyperemesis gravidarum)*
  - ☐ Travail prématuré*
  - ☐ Douleurs sévères au dos*
  - ☐ Anomalie congénitale du fœtus*
  - ☐ Thrombose veineuse profonde
  - ☐ Placenta praevia
  - ☐ Décollement placentaire
  - ☐ Rupture prématurée des membranes
  - ☐ Autre (**précisez**: ___________________________ ***(inclure les complications du fœtus))***
- ☐ Non, vous n’avez pas eu de problèmes de santé pendant cette grossesse.

17. Je vais vous citer une liste de services. Lesquels avez-vous utilisés pendant cette grossesse?

	Oui	*Non*	*Ne sait pas*
**Séance info de la maternité *(expliquer)***	☐	☐	☐
**Cours prénataux de préparation à l'accouchement *(expliquer)***	☐	☐	☐
**Naturopathe ou guérisseur traditionnel**	☐	☐	☐
**NIPT, Test pour la Trisomie ou dépistage de maladies héréditaires ou génétiques (p.ex triple-test, anémie SS ou drépanocytose, maladie méditerranéenne)**	☐	☐	☐
**Échographie**	☐	☐	☐
**Psychologue**	☐	☐	☐
**Kiné prénatale**	☐	☐	☐
**Services sociaux/assistant social**	☐	☐	☐
**Autre (précisez : ____________________)**	☐	☐	☐

18. Dans cette même liste, que je peux vous relire, y avait-il des services que vous n'avez pas utilisés mais que vous auriez aimé utiliser pendant cette grossesse?

(Lisez à voix haute puis cochez <u>toutes</u> les réponses qui s'appliquent)

- ☐ Séance info de la maternité
- ☐ Séances ou cours préparatoires à l'accouchement
- ☐ Naturopathe ou guérisseur traditionnel
- ☐ Test pour la Trisomie
- ☐ Échographie
- ☐ Psychologue
- ☐ Kiné prénatale
- ☐ Aide pour trouver un logement
- ☐ Aide pour accéder aux soins de santé
- ☐ Aide alimentaire
- ☐ Aide financière
- ☐ Autre (**précisez**: ________________________________)
- ☐ Non ***(→ Passez à la Q20)***

19. Pour quelle raison n'avez-vous pas utilisé ou reçu ces services?
(Laissez répondre puis cochez toutes les réponses qui s'appliquent.)

- ☐ Vous ne saviez pas que ces services existaient
- ☐ Vous n'aviez pas le temps
- ☐ Vous n'aviez pas droit à ces services
- ☐ Vous aviez des difficultés administratives
- ☐ Vous ne saviez pas où ces services étaient offerts
- ☐ Vous n'aviez pas cherché de l'aide
- ☐ Les services étaient déjà complets
- ☐ La naissance est arrivée plus tôt que prévu
- ☐ Vous aviez du mal à comprendre le fonctionnement du système de santé
- ☐ Vous aviez peur que ça affecte votre demande d'immigration
- ☐ Barrière linguistique
- ☐ Pas de moyen de transport
- ☐ Problème financier
- ☐ Vous travailliez/ étudiez
- ☐ Vous aviez peur des examens médicaux
- ☐ Vous étiez gênée
- ☐ Vous receviez des conseils ou de l'aide de la part de votre famille ou de vos amis(ies) à la place
- ☐ Ne sait pas
- ☐ Autre (**précisez**:________________________)
- ☐ S/O

---

20. Pendant cette grossesse, aviez-vous l'impression d'avoir suffisamment d'information concernant la grossesse p.ex: les changements de votre corps, votre santé et ce qui est conseillé ou déconseillé faire ?

- ☐ Oui
- ☐ Plus ou moins (**spécifiez ce qui manquait**:____)
- ☐ Non (**spécifiez ce qui manquait**:____)
- ☐ Ne sait pas

---

21. Pendant cette grossesse, aviez-vous l'impression d'avoir suffisamment d'information concernant l'accouchement ?

- ☐ Oui
- ☐ Plus ou moins (**spécifiez ce qui manquait**:____)
- ☐ Non (**spécifiez ce qui manquait**:____)
- ☐ Ne sait pas

---

22. Pendant la grossesse, avez-vous eu l'occasion de discuter avec votre gynéco ou sage-femme du choix d'allaiter ou non votre bébé?

- ☐ Oui
- ☐ Non
- ☐ Vous ne savez pas/ne vous souvenez pas
- ☐ S/O (pas de professionnel de la santé)

23. Pendant la grossesse, comptiez-vous allaiter votre bébé une fois qu'il serait né ?

- ☐ Oui, allaitement maternel exclusif
- ☐ Oui, allaitement mixte (lait maternel et lait en poudre)
- ☐ Non
- ☐ Vous n'étiez pas certaine
- ☐ Vous ne savez plus
- ☐ S/O (pas suivi)

24. Maintenant, allaitez-vous votre bébé ?

- ☐ Oui, allaitement maternel exclusive (au sein ou tiré)
- ☐ Oui, allaitement mixte (lait maternel + lait en poudre/complément)
- ☐ Pas encore (p.ex. bébé prématuré ou autre raison)
- ☐ Non

25. Dans l'ensemble, êtes-vous satisfaite de la prise en charge que vous avez reçu pendant la grossesse?

- ☐ Oui, tout à fait
- ☐ Oui, plus ou moins (**précisez**:____)
- ☐ Non (**précisez** : ____)
- ☐ S/O (pas suivie)

La prochaine série de questions concerne ce qui s'est passé pendant cet ACCOUCHEMENT.

26. Je vais vous citer une série d'interventions médicales. Dites-moi lesquelles ont été utilisées pendant votre accouchement?
(Lire la liste. Si besoin, lire les explications entre parenthèses)

	Oui	*Non*	*Ne sait pas*
**Déclenchement médical du travail** (provoquer les contractions)	☐	☐	☐
**Augmentation du travail** (augmenter l'intensité des contractions existantes par une perfusion d'ocytocine ou rupture de la poche des eaux)	☐	☐	☐
**Utilisation du forceps** (« les cuillères», outil en métal pour faire sortir le bébé)	☐	☐	☐
**Utilisation d'une ventouse** (outil en caoutchouc pour faire sortir le bébé)	☐	☐	☐
**Épisiotomie** (faire une incision/couper pour élargir l'ouverture du vagin)	☐	☐	☐
**Césarienne**	☐	☐	☐
**Anesthésie péridurale ou rachidienne (**anesthésie de la colonne vertébrale pour la douleur durant le travail ou une césarienne)	☐	☐	☐
**Anesthésie générale**	☐	☐	☐
**Autre** ***(specifiez*****: ________________________________)**	☐	☐	☐

→ Si pas de déclenchement médical, passer à Q28

---

27. Pourquoi avez-vous eu un déclenchement médical du travail ?
(Laisser répondre. Si besoin lire la liste. Cochez <u>toutes</u> les options qui s'appliquent)

- ☐ Le bébé n'était pas encore né après le terme
- ☐ Vous aviez rompu la poche des eaux
- ☐ Il y avait un souci avec la santé du bébé
- ☐ Il y avait un souci avec votre santé
- ☐ Le bébé était trop grand ou trop gros
- ☐ Autre: ____________________
- ☐ Vous ne savez pas/ne vous souvenez pas

→ Si pas de césarienne, passer à Q30

---

28. Pourquoi avez-vous eu une césarienne ?
(Laissez répondre puis cochez une réponse)

- ☐ C'était prévu puisque le médecin l'a conseillé pour des raisons médicales
- ☐ C'était prévu, mais vous ne savez pas pourquoi
- ☐ C'était prévu puisque vous l'avez souhaité, ce n'était pas une raison médicale
- ☐ Ce n'était pas prévu, mais le travail durait trop longtemps
- ☐ Ce n'était pas prévu, mais le bébé était en danger
- ☐ Ce n'était pas prévu mais vous étiez en danger
- ☐ Ce n'était pas prévu, et vous ne savez pas pourquoi on vous a fait une césarienne
- ☐ Autre (**précisez:** ____)
- ☐ S/O (accouchement par voie vaginale)

---

29. Aviez-vous démarré le travail avant d'avoir la césarienne ?

- ☐ Oui
- ☐ Non ***(→ sautez à la Q32)***
- ☐ Ne sait pas ***(→ sautez à la Q32)***

---

30. Durant le travail, vous a-t-on permis de bouger et de vous mettre dans des positions confortable de votre choix?
(Lisez à voix haute puis cochez une réponse)

- ☐ Oui, globalement
- ☐ Oui, avant d'avoir la péridurale
- ☐ Oui, mais seulement en ayant insisté
- ☐ Non, pour des raisons médicales
- ☐ Non, pour des raisons que vous ne connaissez pas
- ☐ S/O, le travail n'a pas eu lieu à l'hôpital
- ☐ S/O, il n'y a pas eu de travail

---

31. Durant le travail, étiez-vous satisfaite de la manière dont les sages-femmes ou gynécos vous ont aidée à gérer la douleur?
(Lire à voix haute puis cochez une réponse)

- ☐ Oui
- ☐ Parfois
- ☐ Non
- ☐ S/O, il n'y a pas eu de travail

---

32. Avez-vous pu avoir votre compagnon ou une personne de votre choix avec vous dans la salle d'accouchement pendant le travail et l'accouchement?
(Lisez à voix haute puis cochez une réponse)

- ☐ Oui, pendant le travail et l'accouchement
- ☐ Oui, seulement pendant le travail
- ☐ Oui, seulement pendant l'accouchement
- ☐ Oui, pendant l'accouchement (il n'y a pas eu de travail)
- ☐ Non, pas TOUTES les personnes de votre choix
- ☐ Non, pour des raisons personnelles
- ☐ Non, parce-qu'on ne vous l'a pas permis
- ☐ Vous ne savez pas/ne vous souvenez pas

---

33. Est-ce qu'il y avait des complications médicales durant le travail et l'accouchement- par exemple une déchirure du périnée, une infection, un saignement, un problème avec le bébé ?

- ☐ Oui (**précisez**: ____)
- ☐ Non
- ☐ Ne sait pas

---

34. Dans l'ensemble, êtes-vous satisfaite de la prise en charge que vous avez reçu pendant l'accouchement?

- ☐ Oui, tout à fait
- ☐ Oui, plus ou moins (**précisez**:____)
- ☐ Non (**précisez** : ____)

La prochaine série de questions concerne la période qui s'est découlée depuis la naissance de votre bébé.

35. **Après l'accouchement, sentiez-vous avoir suffisamment d'information sur votre santé?**

- ☐ Oui
- ☐ Plus ou moins (**spécifiez ce qui manquait**:____)
- ☐ Non (**spécifiez ce qui manquait**:____)
- ☐ Ne sait pas

36. **Après l'accouchement, sentiez-vous avoir suffisamment d'information sur la santé de votre bébé?**

- ☐ Oui
- ☐ Plus ou moins (**spécifiez ce qui manquait**:____)
- ☐ Non (**spécifiez ce qui manquait**:____)
- ☐ Ne sait pas

37. **Après l'accouchement, sentiez-vous avoir suffisamment d'information sur comment vous occuper de votre bébé?**

- ☐ Oui
- ☐ Plus ou moins (**spécifiez ce qui manquait**:____)
- ☐ Non (**spécifiez ce qui manquait**:____)
- ☐ Ne sait pas

38. **Dans la première heure après l'accouchement, vous a-t-on posé votre bébé nu sur vous, votre peau directement contre la sienne?**

- ☐ Oui
- ☐ Non (**précisez pourquoi pas**: ____)
- ☐ Ne sait plus

39. **À quel moment une sage-femme ou un gynéco vous a-t-il aidée ou proposé de vous aider à commencer à allaiter?**
(Lire les options puis cochez une réponse)

- ☐ Dans l'heure suivant la naissance
- ☐ Dans les 24 heures suivant la naissance
- ☐ Un autre jour
- ☐ Ils n'ont ni aidé ni proposé d'aider
- ☐ Vous ne savez pas/ne vous souvenez pas
- ☐ S/O (Je n'avais pas besoin d'aide)
- ☐ S/O (Je ne voulais pas allaiter mon enfant)
- ☐ S/O (bébé prématuré ou autre problème de santé)

40. Quand sortirez-vous (ou quand êtes-vous sortie) de la maternité ?

jour__ __ mois__ __

heure :
- ☐ Matin (7h-12h)
- ☐ Après-midi (12h-18h)
- ☐ Soirée (18h-22h)
- ☐ Nuit (22h-7h)
- ☐ Ne sait pas encore
- ☐ Ne se rappelle pas

41. Selon vous, votre séjour à la maternité sera-t-il (était-il) trop court, trop long ou satisfaisant?

- ☐ Trop court
- ☐ Satisfaisant
- ☐ Trop long
- ☐ Vous ne savez pas
- ☐ Vous ne savez pas répondre (sortie pas encore définie)

42. Concernant la sortie de la maternité, vous vous sentez /(sentiez) :
(Lisez à voix haute et cochez une réponse)

- ☐ Tout à fait prête à rentrer à la maison
- ☐ Plutôt prête à rentrer à la maison
- ☐ Pas très prête à rentrer à la maison (**Pourquoi** :____)
- ☐ Pas du tout prête à rentrer à la maison (**Pourquoi** :____)
- ☐ S/O (ne sait pas encore quand sortira)
- ☐ S/O (sortira dans trop longtemps pour dire si elle se sent prête)

43. Dans l'ensemble, êtes-vous satisfaite de la prise en charge que vous avez reçu après l'accouchement ?

- ☐ Oui, tout à fait
- ☐ Oui, plus ou moins (**précisez**:____)
- ☐ Non (**précisez** : ____)

La prochaine série de questions concerne votre expérience <u>globale</u> des soins de santé maternels au cours des 3 périodes : la grossesse, l'accouchement, et après l'accouchement.

44. Lorsque vous y pensez maintenant, est-ce qu'il y avait d'autre conseils, soutien, ou information que vous auriez voulu recevoir?

☐ Non
☐ Oui. → **Précisez** :

____

____

45. Dans l'ensemble, les gynécos et les sages-femmes étaient-elles respectueuses avec vous?
(Lisez à voix haute et cochez une réponse)

☐ Toujours
☐ Souvent
☐ Parfois
☐ Jamais
☐ Ne sait pas

46. Durant votre grossesse, votre accouchement, ou après l'accouchement les sages-femmes ou gynécos vous ont-ils demandé de faire quelque chose avec laquelle vous n'étiez pas d'accord?

☐ Non ***(→ sautez à la Q48)***
☐ Oui
☐ Vous ne savez pas/ne vous souvenez pas ***(→ sautez à la Q48)***

47. Si OUI, qu'est-ce que c'était?

____

☐ S/O

48. Est-ce que, en général, vous préfériez être prise en charge par une femme, un homme ou peu importe?

☐ Femme
☐ Homme ***(→ sautez à la Q50)***
☐ Peu importe ***(→ sautez à la Q52)***
☐ Ne sait pas ***(→ sautez à la Q52)***

49. Avez-vous toujours été prise en charge par une femme ?

- ☐ Oui ***(→ sautez à la Q52)***
- ☐ Non ***(→ sautez à la Q51)***
- ☐ Ne sait pas ***(→ sautez à la Q52)***

50. Avez-vous toujours été prise en charge par un homme?

- ☐ Oui ***(→ sautez à la Q52)***
- ☐ Non
- ☐ Ne sait pas ***(→ sautez à la Q52)***

51. Est-ce que ça vous a gênée?

- ☐ Non
- ☐ Oui
- ☐ Ne sait pas

52. Avez-vous compris les renseignements fournis par les gynécos et les sage-femmes?
(Lisez à voix haute et cochez une réponse)

- ☐ Toujours
- ☐ Souvent
- ☐ Parfois (**Précisez pourquoi**:____) p.ex. à cause de la langue, technicismes
- ☐ Jamais (**Précisez pourquoi**:____) p.ex. à cause de la langue, technicismes
- ☐ Ne sait pas

53. Quelle(s) langue(s) parlez-vous le plus souvent à la maison?_

____

____

→ Si le français est parlé « <u>bien</u> » ou « <u>couramment</u> » (Q5&6) → passer à la Q59

---

54. **Durant la grossesse, l'accouchement ou après l'accouchement, est-ce que vous avez été prise en charge par un gynéco ou une sage-femme qui parlait votre langue ?**
(Lisez à voix haute et cochez une réponse)

- ☐ Non
- ☐ Parfois
- ☐ Oui
- ☐ Ne se souvient plus
- ☐ S/O (pas besoin)

---

55. **Vous a-t-on proposé un service d'interprète ou de traduction?**
(Lisez à voix haute et cochez une réponse)

- ☐ Toujours
- ☐ Souvent
- ☐ Parfois
- ☐ Non
- ☐ Ne se souvient plus
- ☐ S/O

---

56. **Est-ce qu'il y avait quelqu'un avec vous qui parlait votre langue et qui pouvait traduire pour vous?**
(Lisez à voix haute et cochez une réponse)

- ☐ Toujours
- ☐ Souvent
- ☐ Parfois
- ☐ Jamais ***(→ passez à la Q59)***
- ☐ Ne se souvient plus ***(→ passez à la Q59)***
- ☐ S/O

---

57. **Si vous aviez accès à un traducteur, c'était qui?**
(Laissez répondre puis cochez <u>toutes</u> les réponses qui s'appliquent)

- ☐ Mari/femme/conjoint
- ☐ Autre membre de la famille/ami(e)
- ☐ Professionnel(le) de la santé
- ☐ Votre fils ou fille
- ☐ Interprète professionnel
- ☐ Un(e) autre patient(e) ou sa famille/ami(e)
- ☐ Autre (**précisez**: ___________________________
- ☐ S/O

58. **Etiez-vous satisfaite de leur traduction ?**
(Lisez les options à voix haute et cochez une réponse)

- ☐ Oui, en général
- ☐ Oui, plus ou moins
- ☐ Non
- ☐ Vous ne savez pas/ne vous souvenez pas
- ☐ S/O

59. **À votre avis, dans les soins que vous avez reçus, il y a-t-il quelque chose qui pourrait être amélioré ?**

a) Pendant la grossesse

- ☐ Non
- ☐ Oui ***(→ Complétez Q60a)***
- ☐ Vous ne savez pas/ne vous souvenez pas

b) Durant le travail et l'accouchement

- ☐ Non
- ☐ Oui ***(→ Complétez Q60b)***
- ☐ Vous ne savez pas/ne vous souvenez pas

c) Après l'accouchement

- ☐ Non
- ☐ Oui ***(→ Complétez Q60c)***
- ☐ Vous ne savez pas/ne vous souvenez pas

60. **Pouvez-vous me donner des exemples de ce qui pourrait être amélioré?**

a) Pendant la grossesse

____

b) Durant le travail et l'accouchement

____

c) Après l'accouchement

____

61. Qu'est-ce que vous avez particulièrement apprécié dans l'ensemble des soins que vous avez reçus pendant la grossesse, l'accouchement et après l'accouchement?

____

____

☐ Ne sait pas

Toujours concernant l'entièreté des soins que vous avez reçus pendant la grossesse, l'accouchement et après l'accouchement, dites-moi dans quelle mesure les déclarations suivantes sont justes.

62. Les gynécos ou sages-femmes ont pris des décisions sans tenir compte de vos souhaits
(Lisez à voix haute et cochez une réponse)

☐ Toujours
☐ Souvent
☐ Parfois
☐ Jamais
☐ Ne sait pas
☐ S/O (je n'avait pas de souhaits particuliers)
☐ S/O (n'a pas été suivie)

63. Les gynécos et sages-femmes étaient encourageantes et rassurantes
(Lisez à voix haute et cochez une réponse)

☐ Toujours
☐ Souvent
☐ Parfois
☐ Jamais
☐ Ne sait pas
☐ S/O (n'avait pas besoin d'être encouragé ou rassuré)
☐ S/O (n'a pas été suivie)

64. Avez-vous eu l'impression que les gynécos et sage-femmes prenaient vos inquiétudes au sérieux
(Lisez à voix haute et cochez une réponse)

☐ Toujours
☐ Souvent
☐ Parfois
☐ Jamais
☐ Ne sait pas
☐ S/O (n'avait pas d'inquiétudes)
☐ S/O (n'a pas été suivie)

65. Les gynécos et sages-femmes ont passé assez de temps à donner des explications
(Lisez à voix haute et cochez une réponse)

- ☐ Toujours
- ☐ Souvent
- ☐ Parfois
- ☐ Jamais
- ☐ Ne sait pas
- ☐ S/O (n'a pas été suivie)

66. Vous vous sentiez à l'aise de poser des questions à propos de choses que vous ne compreniez pas
(Lisez à voix haute et cochez une réponse)

- ☐ Toujours
- ☐ Souvent
- ☐ Parfois
- ☐ Jamais
- ☐ Ne sait pas
- ☐ S/O (n'avait pas de questions)
- ☐ S/O (n'a pas été suivie)

67. Dans l'ensemble, avez-vous l'impression d'avoir été traitée différemment par les gynécos ou les sage-femmes ? C'est-à-dire…
(Lisez les options à voix haute et cochez une réponse)

- ☐ Vous avez l'impression d'avoir été traitée moins bien que la plupart des gens
- ☐ Mieux que la plupart des gens
- ☐ Pareil que les autres ***(→ Passer à la Q69)***
- ☐ Ne sait pas ***(→ Passer à la Q69***
- ☐ Ne veut pas répondre ***(→ Passer à la Q69***

68. Quelles en étaient les raisons à votre avis?
(Lisez à voix haute puis cochez <u>toutes</u> les réponses qui s'appliquent)

- ☐ Langue ou accent
- ☐ Origine ou couleur de la peau
- ☐ Religion ou culture
- ☐ Niveau scolaire
- ☐ Profession
- ☐ Apparence physique (p.ex. obésité, mutilation génitale)
- ☐ Statut d'immigration (p.ex. sans-papiers) ou statut administratif (p.ex. pas de mutuelle)
- ☐ Autre raison (**précisez**: ________________________)
- ☐ S/O

Les questions suivantes concernent des éventuelles grossesses précédentes

Assurez-vous d'être seules ou dans un environnement intime lorsque vous posez les prochaines questions :

69. Combien de fois avez-vous été enceinte en tout, en comptant cette grossesse? _____ ***(→ si 1, passer à Q79)***

70. Combien de vos grossesses ont été interrompues à cause d'une fausse couche?

_______ *(voir définition fausse couche)*

71. Combien de vos grossesses ont été interrompues par un avortement ou pour des raisons personnelles? _______ *(voir définition IVG)*

72. Combien de vos grossesses ont été interrompues pour des raisons médicales ? _____ *(voir déf. IMG)*

73. Est-ce que vous avez eu un enfant qui est décédé après 5 mois (24 semaines) de grossesse ou dans l'année suivant la naissance? ______ *(voir def. mortalité périnatale et infantile)*

Si oui, c'est arrivé à combien d'enfants?_____

Si oui, c'est arrivé à quel moment :

- ☐ Pendant la grossesse, avant l'accouchement
- ☐ Pendant la naissance
- ☐ Après la naissance

74. Sans compter ce nouveau-né(s), combien de vos autres enfants sont nés prématurés (avant 37 semaines de gestation)? _______*(en considérant uniquement les nés vivants)*

☐ S/O

75. Sans compter ce nouveau-né(s), combien de vos autres enfants sont nés à terme, c'est-à-dire après ou égal à 37 semaines de gestation? ________*(en considérant uniquement les nés vivants)*

☐ S/O

76. En excluant cet accouchement, combien de fois avez-vous accouché en Belgique? __ __

77. Avez-vous eu des problèmes de santé pendant votre (vos) grossesse(s) précédente(s)?

- ☐ Non
- ☐ Oui
- ☐ Ne sait pas
- ☐ S/O (p.ex. grossesse précédente terminé très tôt)

78. Avez-vous eu une césarienne dans le passé?

- ☐ Non
- ☐ Oui
- ☐ Ne sait pas
- ☐ S/O (p.ex. grossesse précédente terminé très tôt)

Les prochaines questions concernent vous et votre famille.

79. Quel est votre situation actuelle? Etes-vous…
(Lire les options et en cocher une)

- ☐ Mariée
- ☐ En union libre ou cohabitation légale (en couple)
- ☐ Célibataire
- ☐ Séparée
- ☐ Divorcée
- ☐ Veuve

80. A propos de votre logement : est-ce que…
(Lire les options à voix haute)

- ☐ Vous êtes locataire ou propriétaire
- ☐ Vous vivez chez la famille
- ☐ Vous êtes hébergée temporairement chez la famille ou chez des amis
- ☐ Vous êtes hébergée en maison d'accueil ou en centre d'accueil, ou hôtel payé par les services sociaux
- ☐ Vous n'avez pas de logement et vous êtes dans la rue ***(→ sautez à la Q86)***
- ☐ Autre (**précisez**: ___________________________________)

81. Lesquelles de ces remarques s'appliquent à votre logement actuel?
(Lisez à voix haute)

	Oui	*Non*	*Ne sait pas*
**C'est un logement social**	☐	☐	☐
**Il est assez grand pour le nombre de gens qui y habitent**	☐	☐	☐
**Il y fait assez chaud l'hiver**	☐	☐	☐
**C'est suffisamment silencieux.**	☐	☐	☐
**Il y a de la moisissure ou de la vermine (ex: cafards, punaises, rats)**	☐	☐	☐
**Il y a de la fumée, p.ex de cigarette ou du chauffage**	☐	☐	☐
**Il est sûr au niveau de sa structure (l'immeuble est solide)**	☐	☐	☐
**Il se situe dans un quartier avec des espaces verts**	☐	☐	☐
**Vous vous sentez en sécurité dans votre quartier**	☐	☐	☐

82. Combien d'enfants habitent avec vous, y compris votre/(vos) nouveau-né(s)? __ __

83. Combien d'adultes habitent avec vous ? __ __ ***(inclure uniquement les personnes qui habitent avec elle la plupart du temps)***

84. Combien de chambres à coucher y-a-t-il dans votre logement ? __ __ ***(Pour studio notez 0)***

85. Combien de pièces de vie y-a-t-il dans votre logement ? C'est à dire salon, salle à manger, ou bureaux (donc en excluant la cuisine, les chambres à coucher, salle de bain, et toilettes) __ __ ***(Pour studio notez 1)***

86. Dans quel pays est née votre mère? ______________

☐ Ne sait pas

87. Dans quel pays est né votre père? ______________

☐ Ne sait pas

88. Dans quel pays est né le père de votre bébé? ______________

☐ Ne sais pas

89. Est-ce que vous avez un lien de parenté sanguin avec le père de votre bébé?

- ☐ Non
- ☐ Oui (**spécifiez** :___________________________)
- ☐ Ne sait pas

90. Qui paie pour vos soins de santé?
(Lire les options et cocher toutes celles qui s'appliquent)

	Oui	*Non*	*Ne sait pas*
**Mutuelle**	☐	☐	☐
**Assurance hospitalisation** (de la mutuelle)	☐	☐	☐
**Assurance maladie privée** (p.ex. de l'employeur)	☐	☐	☐
**Aide médicale urgente/CPAS**	☐	☐	☐
**Assurance gouvernementale pour les réfugiés et les demandeurs d'asile** (Fedasil)	☐	☐	☐
**Vous payez tout vous-même**	☐	☐	☐

91. Bénéficiez-vous d'un tarif préférentiel telle que le Statut BIM, OMNIO ou autre ?
(Laissez répondre et cochez toutes les options qui s'appliquent)

- ☐ Le statut VIPO/BIM/OMNIO (Bénéficiaire d'intervention majorée/remboursement préférentiel de la mutuelle)
- ☐ Autre (**précisez** : ____)
- ☐ Ne sait pas
- ☐ Non

92. Quel est le plus haut niveau scolaire que vous avez terminé ? *(terminé veut dire atteint, diplôme compris s'il y en a un)*
(lisez à voix haute et cochez une réponse)

- ☐ Aucun
- ☐ École primaire
- ☐ Ecole secondaire inférieur (équivalent à ~3 ans de secondaire)
- ☐ École secondaire (équivalent à 6 ou 7 ans de secondaire)
- ☐ Diplôme de haute école ou universitaire (ex: Bachelier, Masters, Doctorat)
- ☐ Autre (**précisez**:___________________________________)

93. <u>Avant votre grossesse</u>, quelle était votre situation professionnelle ?
(Lisez à voix haute et cochez une réponse)

- ☐ Vous travailliez
- ☐ Vous étiez en congé maladie
- ☐ Vous étiez au chômage
- ☐ Vous étiez étudiante
- ☐ Vous étiez au CPAS
- ☐ Vous étiez en incapacité de travail/ invalidité/handicap
- ☐ Vous étiez 'femme au foyer'/ sans revenus/ autre

94. <u>Pendant votre dernier trimestre de grossesse</u>, avant votre congé de maternité, quelle était votre situation professionnelle?
(Lisez à voix haute et cochez une réponse)

- ☐ Vous travailliez
- ☐ Vous étiez employée mais avez été écartée
- ☐ Vous étiez en congé maladie
- ☐ Vous étiez au chômage
- ☐ Vous étiez étudiante
- ☐ Vous étiez au CPAS
- ☐ Vous étiez en incapacité de travail/ invalidité/handicap
- ☐ Vous étiez 'femme au foyer'/ sans revenus/ autre

95. D'après vous, avant votre congé de maternité, dans quel groupe de revenus se situait votre ménage? Pensez au revenu net, c'est-à-dire avant la déclaration fiscale, et en incluant tous les revenus, comme par exemple le revenu d'intégration sociale, allocations familiales, ou chômage ?
(Lire les options et si besoin montrer la plaquette avec les montants)

- ☐ ≤500€
- ☐ 500 à 1000€
- ☐ 1000 à 1500€
- ☐ 1500 à 2000€
- ☐ 2000 à 3000€
- ☐ 3000 à 4000€
- ☐ 4000€
- ☐ S/O (ne sait pas) ***(→ passez à Q97)***
- ☐ S/O (ne veut pas répondre) ***(→ passez à Q97)***

96. Combien de personnes vivent de ce revenu, y compris le nouveau-né? __ __

97. D'où proviennent généralement les revenus de votre ménage ?
(lisez à haute voix et cochez toutes les réponses qui s'appliquent)

- ☐ Emploi déclaré
- ☐ Emploi non déclaré (en black/noir)
- ☐ Activité indépendante
- ☐ Revenu d'intégration sociale (CPAS)
- ☐ Chômage
- ☐ Allocations familiales
- ☐ Indemnité de maladie
- ☐ Indemnité d'invalidité (plus qu'1 an d'incapacité maladie)
- ☐ Allocation pour personnes handicapées
- ☐ Pension
- ☐ Autre (**précisez**: ____________________________)

→ Pour les femmes Belges depuis leur naissance, → sautez à la Q101

Les questions suivantes portent sur votre situation administrative en Belgique. Ces informations nous intéressent parce que nous voulons comprendre l'expérience des femmes migrantes en Belgique pour permettre de mieux adapter les soins de santé. Toute information fournie restera confidentielle, et aucune information ne sera donnée à l'office des étrangers. Vos réponses n'affecteront pas votre demande d'immigration si elle est en cours.

98. Quel statut administratif avez-vous actuellement?
(Lire à voix haute, si besoin montrer la plaquette « statuts d'immigration » et cochez une réponse)

- ☐ Belge
- ☐ Citoyenne de l'Union Européenne
- ☐ Séjour illimité/permanent
- ☐ Réfugiée ou bénéficiaire de la protection subsidiaire
- ☐ Demandeuse d'asile ou de statut de réfugiée
- ☐ Regroupement familial
- ☐ Court séjour (3 mois maximum pour visite familiale, touristique, médicale)
- ☐ Séjour limité aux études/au travail
- ☐ Situation irrégulière/ Sans papiers
- ☐ Victime de traite des êtres humains
- ☐ Autre (veuillez préciser :____________________________)
- ☐ S/O (Ne sait pas) ***(→ passez à Q100)***
- ☐ S/O (Ne veut pas répondre) ***(→ passez à Q100)***

99. Depuis combien de temps avez-vous ce statut?

- ☐ Depuis que vous êtes arrivée en Belgique
- ☐ Autre : ◻ <1 an ◻ 1-5 ans ◻ 6-10 ans ◻ >10 ans ◻ Ne sait pas

100. **Avez-vous l'autorisation légale de travailler en Belgique?**

☐ Oui
☐ Non
☐ Vous ne savez pas

Les prochaines questions abordent votre santé générale.

101. **Souffrez-vous de maladies chroniques, physiques ou mentales? Par ex: diabète, maladie du cœur, dépression, VIH?**

☐ Non
☐ Oui (**Précisez lesquelles**: ____)
☐ Ne sait pas

102. **Combien pesiez-vous avant de tomber enceinte?**

__ __ kg / __ __(livres) __ __(onces)

☐ Ne sait pas ***(→ voir dossier médical)***

103. **Combien pesiez-vous avant d'accoucher?**

__ __ kg / __ __(livres) __ __(onces)

☐ Ne sait pas ***(→ voir dossier médical)***

104. **Combien mesurez-vous?**

__ (m) __ __((cm)/ __ (pieds) __ __((pouces)

☐ Ne sait pas ***(→ voir dossier médical)***

Nous avons quelques questions au sujet de la planification de cette grossesse.

105. Au moment où vous êtes tombée enceinte de ce bébé, vouliez-vous tomber enceinte?
(Lire les options à voix haute et cocher une réponse)

- ☐ Oui
- ☐ Non ***(→Passez à Q107)***
- ☐ Incertaine ***(→Passez à Q107)***
- ☐ S/O (Ne veut pas répondre) ***(→Passez à Q107)***

106. Est-ce que la conception de ce bébé a été médicalement assistée (c.a.d. vous avez fait recours à la PMA)?

- ☐ Oui ***(→Passez à Q110)***
- ☐ Non ***(→Passez à Q110)***
- ☐ Ne veut pas répondre ***(→Passez à Q110)***

107. Utilisiez-vous une méthode contraceptive pour empêcher une grossesse au moment où vous êtes tombée enceinte (par exemple préservatif, pilule ou autre)?
(Si besoin, consultez la liste à la Q108 pour des exemples)

- ☐ Oui
- ☐ Non ***(→Passez à Q109)***
- ☐ Ne sait pas ***(→Passez à Q109)***

108. Qu'est-ce que vous utilisiez?
(Laissez répondre, cochez <u>toutes</u> les réponses qui s'appliquent, puis passez à Q110)

- ☐ Préservatif
- ☐ Pilule contraceptive
- ☐ Dispositif intra-utérin (DIU)
- ☐ Diaphragme
- ☐ Contraceptif injectable Depo-Provera
- ☐ Insertion sous la peau du bras (Norplant)
- ☐ Allaitement
- ☐ Rétraction ("pull-out")
- ☐ Observation du cycle mensuel
- ☐ Vous pensiez que vous ou votre partenaire étiez sterile
- ☐ Abstinence
- ☐ Autre (**précisez** : ________________________________)
- ☐ S/O

109. **Si vous n'utilisiez pas de contraception, pourquoi?**
(Laissez répondre puis cochez toutes les réponses qui s'appliquent)

- ☐ Aucun accès à une clinique ou à un fournisseur de soins de santé
- ☐ Effets secondaires
- ☐ Vous n'aviez pas les moyens
- ☐ Raisons religieuses
- ☐ Votre mari/votre famille vous l'ont interdit
- ☐ Autre (**précisez** : ___________________________________
- ☐ S/O

Pour conclure, nous avons quelques questions à propos de vos habitudes et votre santé

110. **Dans l'année qui a précédé cette grossesse, avez-vu fumé du tabac?**
(lisez à voix haute les options et cochez en une)

- ☐ Non ***(→ Passez à Q112)***
- ☐ Oui, parfois
- ☐ Oui, chaque jour ou presque chaque jour
- ☐ Oui, mais j'ai arrêté de fumer pendant l'année qui a précédé la grossesse ***(→ Passez à Q112***

111. **Durant cette grossesse, est-ce que ça vous est arrivé de fumer du tabac?**
(lisez à voix haute les options et cochez en une)

- ☐ Non
- ☐ Oui, parfois
- ☐ Oui, chaque jour ou presque chaque jour
- ☐ Oui, mais j'ai arrêté de fumer pendant la grossesse

112. **Durant votre grossesse, est-ce que ça vous est arrivé de consommer des boissons alcoolisées?**
(Lisez à voix haute et cochez une réponse)

- ☐ Jamais
- ☐ Rarement (≤ 1 boisson/mois)
- ☐ Parfois (entre 2 boissons/mois et 1 boisson/semaine)
- ☐ Souvent (≥ 2 boissons/semaine)

113. **Au moins un mois avant que vous tombiez enceinte, avez-vous pris des vitamines pour la grossesse ou de l'acide folique?**
(Si oui, lisez à voix haute les options avec « oui » et cochez-en une)

- ☐ Oui, presque chaque jour ***(→ Passez à Q115)***
- ☐ Oui, de temps en temps
- ☐ Non
- ☐ Ne sait pas ***(→ Passez à Q115)***

114. Si « non » ou seulement « de temps en temps », pourquoi?
(Laissez répondre et cochez toutes les réponses qui s'appliquent)

- ☐ On ne lui a pas dit d'en prendre/ ne savait pas qu'il fallait en prendre
- ☐ N'en avait pas besoin
- ☐ Ne savait pas à quoi ça servait
- ☐ Oubli
- ☐ Effets secondaires (p.ex; nausée)
- ☐ N'avait pas les moyens de l'acheter
- ☐ Ne pouvait pas en trouver
- ☐ N'avait pas planifié la grossesse
- ☐ Autre (**précisez** : ____)
- ☐ S/O

115. Durant votre grossesse, avez-vous pris des vitamines pour la grossesse ou de l'acide folique?
(Si oui, lisez à voix haute les options avec « oui » et cochez-en une)

- ☐ Oui, presque chaque jour ***(→ Fin du questionnaire)***
- ☐ Oui, de temps en temps
- ☐ Non
- ☐ Ne sait pas ***(→ Fin du questionnaire)***

116. Si 'non' ou seulement 'de temps en temps', pourquoi?
(Laissez répondre et cochez toutes les réponses qui s'appliquent)

- ☐ On ne lui a pas dit d'en prendre/ ne savait pas qu'il fallait en prendre
- ☐ N'en avait pas besoin
- ☐ Ne savait pas à quoi ça servait
- ☐ Oubli
- ☐ Effets secondaires (p.ex; nausée)
- ☐ N'avait pas les moyens de l'acheter
- ☐ Ne pouvait pas en trouver
- ☐ Autre (**précisez** : ____)
- ☐ S/O

Ceci conclue notre entrevue. Je vous remercie beaucoup pour votre temps et pour avoir partagé votre vécu aujourd'hui.
Avez-vous des questions ?
Merci, je vous souhaite une très bonne continuation.

<u>Definitions</u>:

Fausse-couche: decès de foetus < 24 semaines ou 500g

IVG : interruption volontaire de grossesse : interruption volontaire (en Belgique <12 semaines de gestation, autres pays <12 voir jusqu'à 24 semaines)

IMG : interruption médicale de grossesse : interruption pour raisons médicales (santé de la maman ou du bébé) jusqu'à terme

Mortalité périnatale et infantile: décès de foetus > 24 semaines de gestation ou 500g, décès à la naissance, ou dans la semaine suivant la naissance. Mortalité infantile : décès dans l'année suivant la naissance
