# Supplementary material for: Country of birth as a potential determinant of inadequate antenatal care use among women giving birth in Brussels. A cross-sectional study
Source: PLoS One. 2022 Apr 15;17(4):e0267098. doi: 10.1371/journal.pone.0267098 (PMC9012396; doi:10.1371/journal.pone.0267098)
Supplement: S2 File — English version. (PDF) [file pone.0267098.s004.pdf]

Migrant Friendly Maternity Care Questionnaire- adapted version

Adapted MFMCQ – Brussels – English - December 2018

Introduction

As explained in the information document that you have received, I am working with a research team that aims to understand the care given to pregnant women. I will be asking questions about your experience with healthcare during your pregnancy, birth, and the period after your baby/(babies) was born. Some questions concern your health and your social background. Feel free to interrupt me if you have any questions or want me to repeat a question. All the information you give will remain confidential. You may stop your participation in the study at any time, and you may choose not to answer any questions you don't feel comfortable with.
Do you have any questions before we begin?
Alright, let's get started!

**Identification number:**		**Interviewer code:**	
**Current nationality:**		**Interview date:**	__/__/____
**Delivery hospital:**		**Day after delivery:**	D0, D1, D2, D3, D___
**Interview mode:**	Hosp□ Tel□ Home□	**Interview language:**	
**Time start:**		**Interpreter:**	Yes □ No □
**Time end:**			

To start with, I will ask some general questions on your background

1. Which country were you born in?

- □ Belgium
- □ Other : ______________

2. What was your nationality when you were born?

- □ Belgium
- □ Other ____________________

3. How long have you lived in Belgium?

__ __ (years) ___ __ (months) __ __ (weeks)

(TOTAL amount of time the mother lived in this country)

4. How old are you?

__ __ years

5. What's your level of French ?
(read aloud and check one option in each line. Remember the answer to "oral" for later.)

	Fluent	*Good*	*With difficulty*	*Not at all*
Oral (speaking et understanding)	☐	☐	☐	☐
Reading and Writing	☐	☐	☐	☐

6. What's your level of Dutch ?
(read aloud and check one option in each line. Remember the answer to "oral" for later.)

	Fluent	*Good*	*With difficulty*	*Not at all*
Oral (speaking et understanding)	☐	☐	☐	☐
Reading and Writing	☐	☐	☐	☐

The following questions concern your recent PREGNANCY

7. During this pregnancy, did you consult a doctor, midwife, or other healthcare professional?

☐ Yes **(in which country:** ☐ Belgium ☐ elsewhere**)**
☐ No **(why not? ________________________) (*→Skip to Q16*)**

8. Has your pregnancy mainly been followed-up by an obstetrician, a midwife, or both?

☐ Gynaecologist/ Obstetrician
☐ Midwife
☐ Both
☐ Other (**specify**: ____________)
☐ N/A

9. How many weeks pregnant were you when you first consulted for this pregnancy?
(Don't count a visit that was ONLY for a pregnancy test)

☐ First 2 months (4-9 weeks) **(*→Skip to Q11*)**
☐ 3rd months (10-13 weeks) **(*→Skip to Q11*)**
☐ 4th months (14-18 weeks)
☐ 5th months (19-23 weeks)
☐ 6th months (24-28 weeks)
☐ After the 6th month (29+ weeks)
☐ Don't know **(*→Skip to Q11*)**
☐ N/A no care received

10. Why did you not consult earlier?
(read aloud and check one)

- ☐ You didn't feel the need
- ☐ You didn't know who to contact
- ☐ It was difficult to get an appointment with an obstetrician or a midwife for administrative reasons (e.g; problem with health insurance, or urgent medical care)
- ☐ An earlier appointment was not available
- ☐ You didn't know that you were pregnant
- ☐ Other reason:____________________________

11. Did you encounter any difficulties to get an appointment with an obstetrician or a midwife?

- ☐ No
- ☐ Yes (**Please specify:** ____)
- ☐ Don't know

12. Overall, how many visits did you have with an obstetrician or midwife for this pregnancy, excluding appointments for ultrasound scans only?

- ☐ 1-2 visits
- ☐ 3-6 visits
- ☐ 7-9 visits
- ☐ ≥10 visits
- ☐ Don't know
- ☐ N/A no care received

13. Would you have liked to have more appointments with your obstetrician or midwife during this pregnancy?

- ☐ Yes
- ☐ No
- ☐ Don't know
- ☐ N/A no care received

14. During this pregnancy, were you followed-up by the same obstetrician or midwife?

- ☐ Yes, always **(*→Skip to Q16*)**
- ☐ Yes, most of the time **(*→Skip to Q16*)**
- ☐ No
- ☐ Don't know
- ☐ N/A no care received **(*→Skip to Q16*)**

15. Did you have the impression that there was a good continuity in you care? In other words, did the obstetricians or midwives you saw exchange information and work well together?

- ☐ Yes, mostly
- ☐ Yes, more or less
- ☐ No
- ☐ Don’t know
- ☐ N/A no care received

16. Did you or your baby have any health issues during this pregnancy?

☐ Yes ***(allow mother to answer and check all that apply. Then, ask specifically about items with *)***

- ☐ Anaemia*
- ☐ High blood pressure* (either preceding pregnancy or developed during pregnancy)
- ☐ Pre-eclampsia*
- ☐ Gestational diabetes*
- ☐ Urinary infection*
- ☐ Depression*
- ☐ Severe nausea and vomiting (hyperemesis gravidarum)*
- ☐ Preterm labour*
- ☐ Severe back pain*
- ☐ Congenital anomaly*
- ☐ Deep vein thrombosis (DVT)
- ☐ Placenta praevia
- ☐ Placental abruption
- ☐ Premature rupture of membranes
- ☐ Other *(**specify**:* ________________________ ***(including foetal complications))***

☐ No, you did not have any health issues during this pregnancy

17. I’m going to read out a list of services. Which of the following did you use during this pregnancy?

	Yes	*No*	*Don’t know*
**Maternity ward information session *(explain)***	☐	☐	☐
**Prenatal classes to prepare for childbirth *(explain)***	☐	☐	☐
**Naturopath or traditional healer**	☐	☐	☐
**NIPT, Trisomy screening test or screening for hereditary diseases (e.g. triple-test, sickle cell anaemia)**	☐	☐	☐
**Ultrasound scan**	☐	☐	☐
**Psychological support**	☐	☐	☐
**Prenatal physiotherapy**	☐	☐	☐
**Social support services**	☐	☐	☐
**Other (specify : ________________________________)**	☐	☐	☐

18. In this same list, which I can read out again, were there services that you didn't use but that you would have liked to use during this pregnancy?
(Read aloud and check all that apply)

- ☐ Delivery ward information session
- ☐ Prenatal classes to prepare for childbirth
- ☐ Naturopath or traditional healer
- ☐ Trisomy screening test
- ☐ Ultrasound scan
- ☐ Psychologist
- ☐ Prenatal physiotherapy
- ☐ Help finding accomodation
- ☐ Help accessing healthcare services
- ☐ Food banks
- ☐ Financial help
- ☐ Other **(Specify: ______________________________________)**
- ☐ No **(→*Skip to Q20)***

19. For what reasons did you not use or receive these services?
(Allow mother to answer and check all that apply.)

- ☐ You didn't know these services existed
- ☐ You didn't have the time
- ☐ You were not eligible for these services
- ☐ You had administrative difficulties
- ☐ You did not know where these services were offered
- ☐ You didn't seek help
- ☐ The services were already full
- ☐ The baby was born earlier than expected
- ☐ You had difficulties understanding how the healthcare system works
- ☐ You fear that your immigration application would be affected
- ☐ Linguistic barrier
- ☐ Lack of means of transport
- ☐ Financial problems
- ☐ You were working/ studying
- ☐ Fear of medical tests or examinations
- ☐ You were embarassed
- ☐ You got advice & help from family and friends instead
- ☐ Don't know
- ☐ Other **(specify :____)**
- ☐ N/A

20. During this pregnancy, did you feel that you had sufficient information regarding pregnancy, like the changes affecting your body, your health, and what is advised or better avoided during pregnancy?

- ☐ Yes
- ☐ More or less (**specify what was missing:**____)
- ☐ No (**specify what was missing**: ____)
- ☐ Don't know

21. During this pregnancy, did you feel that you had enough information about giving birth?

- ☐ Yes
- ☐ More or less (**specify what was missing:**____)
- ☐ No (**specify what was missing:** ____)
- ☐ Don't know

22. During this pregnancy, did you have the opportunity to speak with a healthcare professional about the choice of breastfeeding or not?

- ☐ Yes
- ☐ No
- ☐ Don't know/ don't remember
- ☐ N/A (no contact with healthcare professionals)

23. During the pregnancy, did you plan to breastfeed your baby once (s)he would be born?

- ☐ Yes, exclusive breastfeeding
- ☐ Yes, partly breastfeeding and partly bottle-feeding
- ☐ No
- ☐ You weren't sure
- ☐ You don't remember
- ☐ N/A no care received

24. Now, do you breastfeed your baby ?

- ☐ Yes, exclusive breastfeeding
- ☐ Yes, partly breastfeeding and partly bottle-feeding
- ☐ Not yet (baby born premature or other reason)
- ☐ No

25. Overall, are you satisfied with the care you received during your pregnancy?

- ☐ Yes, completely satisfied
- ☐ Yes, more or less (**please specify:** ____)
- ☐ No (**please specify:** ____)
- ☐ N/A no care received

The next questions concern your recent LABOUR and the BIRTH of your baby/babies.

26. I'm going to read out a series of medical interventions. Tell me which have been used during labour or birth.
(Read the list. If necessary, read the explanations between brackets)

	Yes	*No*	*Don't know*
**Induction of labour** (making your contractions start)	☐	☐	☐
**Augmentation of labour** (making contractions stronger and more frequent with intravenous oxytocin or breaking your waters)	☐	☐	☐
**Use of forceps** (metal tool to remove the baby)	☐	☐	☐
**Use of ventouse** (suction tool to remove the baby)	☐	☐	☐
**Episiotomy** (cut near the opening of the vagina)	☐	☐	☐
**Caesarean section**	☐	☐	☐
**Epidural anesthesia** (anaesthesia of the spine for labour pain or for caesarean section)	☐	☐	☐
**General anaesthesia**	☐	☐	☐
**Other *(specify*: ____)**	☐	☐	☐

→ If no induction of labour skip to Q28

27. Why was your labour induced?
(Let the participant answer. Read out the list if need be, and check all answers that apply)

- ☐ The baby wasn't yet born after term
- ☐ Your waters had broken
- ☐ There was a worry with the baby's health
- ☐ There was a worry with your health
- ☐ The baby was too big
- ☐ Other: ________________________
- ☐ You don't know/you don't remember

→ If didn't have a caesarean section, skip to Q30

28. Why did you have a caesarean section?
(Allow mother to answer and check one)

- ☐ It was planned because the doctor suggested it for medical reasons
- ☐ It was planned but you don't know why
- ☐ It was planned because you wanted it, but not for a medical reason
- ☐ It was not planned, but your labour was taking too long
- ☐ It was not planned, but the baby was in danger
- ☐ It was not planned, but you were in danger
- ☐ It was not planned, and you don't know why it happened
- ☐ Other (**specify**:____)
- ☐ N/A (delivered vaginally)

---

29. Were you in labour before you had the caesarean section?

- ☐ Yes
- ☐ No (→***Skip to Q32)***
- ☐ Don't know (→***Skip to Q32)***

---

30. During labour, were you allowed to move and choose comfortable positions?
(Read aloud and check one)

- ☐ Yes, overall
- ☐ Yes, before having the epidural
- ☐ Yes, but only after having insisted
- ☐ No, for medical reasons
- ☐ No, for reasons you don't know
- ☐ N/A, your labour didn't take place in hospital
- ☐ N/A, you had no labour

---

31. During labour, were you satisfied with how midwives or obstetricians helped you manage your pain?
(Read aloud and check one)

- ☐ Yes
- ☐ Sometimes
- ☐ No
- ☐ N/A, there was no labour

---

32. Were you allowed to be accompanied by your partner or a person of your choice during labour and birth?
(Read aloud and check one)

- ☐ Yes, during labour and birth
- ☐ Yes, but only during labour
- ☐ Yes, but only during birth
- ☐ Yes, during birth (there was no labour)
- ☐ No, not ALL the people of your choice
- ☐ No, for personal reasons
- ☐ No, because it wasn't allowed
- ☐ You don't know/ can't remember

---

33. Were there any medical complications during labour and birth ? For example : perineal tear, infection, haemorrhage, problems with the baby.

- ☐ Yes (***specify***: ____)
- ☐ No
- ☐ Don't know

34. Overall, were you satisfied with the care you received during labour and birth?

- ☐ Yes, completely
- ☐ Yes, more or less (**specify**:____)
- ☐ No (**specify**: ____)

The next set of questions concerns the time since your baby was born.

35. A<u>fter the birth of your baby/(babies)</u>, did you feel that you had enough information about your health?

- ☐ Yes
- ☐ More or less (**specify what was missing:** ________________________________)
- ☐ No (**specify what was missing:**________________________________)
- ☐ Don't know

36. A<u>fter the birth of your baby</u>, did you feel that you had enough information about you baby's health?

- ☐ Yes
- ☐ More or less (**specify what was missing:** ________________________________)
- ☐ No (**specify what was missing:**________________________________)
- ☐ Don't know

37. A<u>fter the birth of your baby</u>, did you feel that you had enough information about how to take care of you baby?

- ☐ Yes
- ☐ More or less (**specify what was missing:** ________________________________)
- ☐ No (**specify what was missing:**________________________________)
- ☐ Don't know

38. <u>In the first hour</u> after birth, were you given your baby to hold skin-to-skin (with the baby's bare skin directly next to your bare skin)?

- ☐ Yes
- ☐ No (**specify why not**: ________________________________)
- ☐ Don't remember

39. When did your heathcare professional help you or offer to help you start breastfeeding?
(Allow mother to answer and check one)

- ☐ In the first hour after birth
- ☐ In the 24 hours following birth
- ☐ Another day
- ☐ They did not help or offer help
- ☐ Don't know/ don't remember
- ☐ N/A (didn't need help)
- ☐ N/A (did not want to breastfeed)
- ☐ N/A (premature baby or other health problem)

---

40. When will you go home from hospital (or when did you leave hospital)?

day__ __ month__ __

time:

- ☐ Morning (7h-12h)
- ☐ Afternoon (12h-18h)
- ☐ Evening (18h-22h)
- ☐ Night (22h-7h)
- ☐ Don't know yet
- ☐ Don't remember

---

41. Do you find your hospital stay too short, too long, or about right?

- ☐ Too short
- ☐ About right
- ☐ Too long
- ☐ Don't know
- ☐ N/A can't answer (don't know when leaving)

---

42. Concerning leaving hospital and returning back home, do (did) you feel:
(read aloud and check one)

- ☐ Completely ready to return home
- ☐ Fairly ready to return home
- ☐ Not very ready to return home (**Why**: ______________________________)
- ☐ Not at all ready to return home (**Why**: -_____________________________)
- ☐ N/A (don't know yet when will leave hospital)
- ☐ N/A (will leave in too long to say whether feel ready or not)

---

43. Overall, are you satisfied with the care you received after having given birth?

- ☐ Yes, completely
- ☐ Yes, more or less (**specify:** ______________________________)
- ☐ No (**specify:** ______________________________)

The next series of questions concerns your overall experience with the maternity healthcare services over the 3 periods: pregnancy, birth, and after birth.

44. Thinking about it now, was there any other advice, support, or information you wish you had received?

- ☐ No
- ☐ Yes. → **Specify :**

____

____

45. Overall, were the midwives and obstetricians respectful?
(read aloud and check one)

- ☐ Always
- ☐ Often
- ☐ Sometimes
- ☐ Never
- ☐ Don't know

46. During your pregnancy, labour, or birth, did the healthcare professionals ask you to do something that you were not ok with?

- ☐ No ***(→skip to Q48)***
- ☐ Yes
- ☐ Don't know/ Don't remember ***(→skip to Q48)***

47. If yes, what was it?

____

- ☐ N/A

48. Overall, did you prefer having a healthcare professional that was female, male, or were you ok with either?

- ☐ Female
- ☐ Male ***(→ skip to Q50)***
- ☐ Either ***(→ skip to Q52)***
- ☐ Don't know ***(→ skip to Q52)***

49. Were you always followed-up by a female healthcare professional?

- ☐ Yes ***(→ skip to Q52)***
- ☐ No ***(→ skip to Q51)***
- ☐ Don't know ***(→ skip to Q52)***

50. Were you always followed-up by a male healthcare professional?

- ☐ Yes ***(→ skip to Q52)***
- ☐ No
- ☐ Don't know ***(→ skip to Q52)***

---

51. Has this embarrassed or bothered you?

- ☐ No
- ☐ Yes
- ☐ Don't know

---

52. Did you understand the information provided by healthcare professionals?

- ☐ Always
- ☐ Often
- ☐ Sometimes (**Specify why**:____) e.g. due to language, jargon
- ☐ Never (**Specify why**:____) e.g. due to language, jargon
- ☐ Don't know

---

53. What language(s) do you speak most often at home?

____

____

→ If French or Dutch is spoken « good » or « fluently » (Q5&6) → skip to Q59

---

54. During your pregnancy, delivery, or after having given birth, were you cared for by an obstetrician or midwife who spoke your language?
(read aloud and check one)

- ☐ No
- ☐ Sometimes
- ☐ Yes
- ☐ Can't rememebr
- ☐ N/A (no need)

---

55. Did the healthcare professionals offer you an interpreting service?
(read aloud and check one)

- ☐ Always
- ☐ Often
- ☐ Sometimes
- ☐ No
- ☐ Can't remember
- ☐ N/A

56. How often was there someone with you who spoke your language and could interpret for you?
(read aloud and check one)

- ☐ Always
- ☐ Often
- ☐ Sometimes
- ☐ Never ***(→ skip to Q59)***
- ☐ Can't remember ***(→ skip to Q59)***
- ☐ N/A

57. If you had someone to interpret for you, who was it?
(Read aloud and check all that apply)

- ☐ Husband/wife/partner
- ☐ Other family member/friend
- ☐ Healthcare practitioner
- ☐ You child
- ☐ Professional interpreter
- ☐ Another patient or patient's family member/friend
- ☐ Other **(specify: _________________________)**
- ☐ N/A

58. Were you satisfied with their interpretation?
(read aloud and check one)

- ☐ Yes, mostly
- ☐ Yes, more or less
- ☐ No
- ☐ Don't know/can't remember
- ☐ N/A

59. In your opinion, is there anything that could be improved in the care that you received?

a) During pregnancy
- ☐ No
- ☐ Yes ***(→ fill in Q60a)***
- ☐ Don't know/ can't remember

b) During labour and birth
- ☐ No
- ☐ Yes ***(→ fill in Q60b)***
- ☐ Don't know/ can't remember

c) After birth
- ☐ No
- ☐ Yes ***(→ fill in Q60c)***
- ☐ Don't know/ can't remember

60. Can you give me an example of what could be improved?

a) During pregnancy

____

b) During delivery and birth

____

c) After birth

____

61. Please describe anything about your care during pregnancy, birth, or after birth that you are particularly happy with?

____

____

- ☐ Don’t know

The next section still concerns the overall care you received during pregnancy, delivery, and after delivery. Please tell me how often the following statements were true.

62. The obstetricians or midwives made decisions without your wishes being taken into account ***(read aloud and check one)***

- ☐ Always
- ☐ Often
- ☐ Sometimes
- ☐ Never
- ☐ Don’t know
- ☐ N/A (didn’t have any particular wishes)
- ☐ N/A (didn’t receive any care)

63. The obstetricians and midwives were encouraging and reassuring ***(read aloud and check one)***

- ☐ Always
- ☐ Often
- ☐ Sometimes
- ☐ Never
- ☐ Don’t know
- ☐ N/A (didn’t need to be encouraged or reassured)
- ☐ N/A (didn’t receive any care)

64. You felt that obstetricians and midwives took your worries seriously
(read aloud and check one)

- ☐ Always
- ☐ Often
- ☐ Sometimes
- ☐ Never
- ☐ Don't know
- ☐ N/A (didn't have any worries)
- ☐ N/A (didn't receive any care)

65. The obstetricians and midwives spent enough time providing explanations.
(read aloud and check one)

- ☐ Always
- ☐ Often
- ☐ Sometimes
- ☐ Never
- ☐ Don't know
- ☐ N/A (didn't receive any care)

66. You felt comfortable asking about things you did not understand
(read aloud and check one)

- ☐ Always
- ☐ Often
- ☐ Sometimes
- ☐ Never
- ☐ Don't know
- ☐ N/A (didn't have questions)
- ☐ N/A (didn't receive any care)

67. Overall, do you feel that you were treated differently to other people by obstetricians or midwives? That is…
(read aloud and check one)

- ☐ You felt you were treated worse than most people
- ☐ You felt treated better than most people
- ☐ Same as other people ***(→ skip to Q69)***
- ☐ Don't know ***(→ skip to Q69)***
- ☐ Don't want to answer ***(→ skip to Q69)***

68. What were the reasons in your opinion?
(Read aloud and check all that apply)

- ☐ Language or accent
- ☐ Origin or skin colour
- ☐ Religion or culture
- ☐ Level of education
- ☐ Profession
- ☐ Physical appearance (e.g. obesity, female genital mutilation)
- ☐ Immigration status (e.g. undocumented) or administrative status (e.g. no health insurance)
- ☐ Other reason **(specify: ________________)**
- ☐ N/A

The following questions concern any previous pregnancy you may have had

Be sure no one else is present or you are in an intimate environment when you ask the following questions:

69. How many pregnancies have you had in total, including this pregnancy? ______ ***(→ if 1, skip to Q79)***

70. How many pregnancies ended in miscarriage? _______ *(see definition of miscarriage)*

71. How many pregnancies were terminated by a termination of pregnancy (T.O.P.) or for personal reasons? _______ *(see definition of TOP)*

72. How many pregnancies were terminated for medical reasons? ________ *(see defintion of MTOP)*

73. Have you had a baby who died after 5 months (24 weeks) of pregnancy or in the year following its birth? _____ *(see definition of perinatal and infant mortality)*

If yes, how many children did this happen to? ____

If yes, when did this happen:
- ☐ During pregnancy, before birth
- ☐ During birth
- ☐ After birth

74. Excluding this newborn(s), how many of your other children were born before 37 completed weeks of gestation? _______ *(considering only live births)*

- ☐ N/A

75. Excluding this newborn(s), how many of your other children were born at term, that is after 37 completed weeks of gestation? ________ *(considering only live births)*

- ☐ N/A

76. Excluding this delivery, how many times did you give birth in Belgium ? __ __

77. Did you have any health problems in your previous pregnancie(s)?

- ☐ No
- ☐ Yes
- ☐ Don't know
- ☐ N/A (e.g. previous pregnancie(s) ended very early)

78. Did you have a caesarean-section in the past?

- ☐ No
- ☐ Yes
- ☐ Don't know
- ☐ N/A (e.g. previous pregnancie(s) ended very early)

The next questions concern you and your family.

79. What is your current situation? Are you…
(read aloud and check one)

- ☐ Married
- ☐ Consensual union or cohabitation (in a couple)
- ☐ Single
- ☐ Separated
- ☐ Divorced
- ☐ Widowed

80. Concerning your accommodation: …
(read aloud and check one)

- ☐ Do you rent or own your accommodation
- ☐ Do you live at your family's place
- ☐ Are your temporarily living at your family's or friend's place
- ☐ Are you staying in a reception centre, shelter, or in a hotel paid for by social services
- ☐ You don't have any accommodation and you are homeless ***(→ skip to Q86)***
- ☐ Other **(specify: ________________________________)**

81. Which statements apply to your current accommodation?
(read aloud)

	Yes	*No*	*Don't know*
**It is social housing**	☐	☐	☐
**It is large enough for the number of people living there**	☐	☐	☐
**It is warm enough in the winter**	☐	☐	☐
**It is quiet enough**	☐	☐	☐
**There is mould or pests (e.g. insects, bedbugs, rats)**	☐	☐	☐
**There is indoor smoke, e.g. from cigarette or heating**	☐	☐	☐
**It is structurally safe (the building is strong)**	☐	☐	☐
**It is in a neighbourhood with green spaces**	☐	☐	☐
**You feel safe in your neighbourhood**	☐	☐	☐

82. How many children live with you, including your newborn baby/babies? __ __

83. How many adults live with you? __ __ *(include only the people who live with her most of the time)*

84. How many bedrooms are there? __ __ *(For a studio write 0)*

85. How many rooms are there in your accommodation? That is living rooms, diners, or offices (so excluding the kitchen, bedrooms, bathrooms, and toilets) __ __ *(For a studio write 1)*

86. In which country was your mother born? ____________

☐ Don't know

87. In which country was your father born? _____________

☐ Don't know

88. In which country was the father or your baby born? _______________

☐ Don't know

89. Is the father of your baby blood related to you?

☐ No
☐ Yes **(specify:___________________________)**

90. Who pays for your medical care?
(Read aloud and check all that apply)

	Yes	*No*	*Don't know*
**Public health insurance** *("mutuelle")*	☐	☐	☐
**Hospitalisation insurance** *(of the mutuelle)*	☐	☐	☐
**Private health insurance**	☐	☐	☐
**Urgent Medical Care/CPAS** *(access to care through social services)*	☐	☐	☐
**Insurance through the Federal Agency for the reception of asylum seekers** *(Fedasil)*	☐	☐	☐
**You pay for all the care yourself**	☐	☐	☐

91. Do you benefit from a preferential rate such as the « Statut BIM », « OMNIO», or something similar?
(check all that apply)

- ☐ BIM/OMNIO/VIPO status (beneficiary of increased reimbursement from the mutuelle)
- ☐ Other **(specify : ____)**
- ☐ Don't know
- ☐ No

92. What is the highest level of education you have completed? *(completed means achieved, including the diploma where relevant)*
(read aloud and check one)

- ☐ None
- ☐ Primary school
- ☐ Lower secondary school (equivalent to ~3 years of secondary education)
- ☐ Secondary school/high school/ A-levels (equivalent to ~6 or 7 years of secondary education)
- ☐ University diploma or diploma from a "haute école" (ex: Bachelor, Masters, PhD)
- ☐ Other **(specify:________________________________)**

93. Before your pregnancy, what was your professional situation?
(read aloud and check one)

- ☐ Working
- ☐ On sick leave
- ☐ Unemployed
- ☐ Student
- ☐ Recipient of social welfare of last resort (CPAS)
- ☐ Work incapacity/disability
- ☐ No income/'housewife'/other

94. **<u>During your last trimester of pregnancy</u>, before your maternity leave, what was your professional situation?**
(read aloud and check one answer)

- ☐ Working
- ☐ You were employed but on pregnancy leave
- ☐ On sick leave
- ☐ Unemployed
- ☐ Student
- ☐ Recipient of social welfare of last resort (CPAS)
- ☐ Work incapacity/disability
- ☐ No income/'housewife'/other

95. **According to you, before your maternity leave, which range of incomes did your household belong to? Think of the net income, that is before the tax return, and including all income, such as social integration income, child benefits, or unemployment allowance.**
(Read aloud, and if necessary show the card with the values)

- ☐ ≤500€
- ☐ 500 to 1000€
- ☐ 1000 to 1500€
- ☐ 1500 to 2000€
- ☐ 2000 to 3000€
- ☐ 3000 to 4000€
- ☐ ≥ 4000€
- ☐ N/A (don't know) ***(→ skip to Q97)***
- ☐ N/A (doesn't want to answer) ***(→ skip to Q97)***

96. **How many people does this income support, including your newborn baby? __ __**

97. **Where does your household income generally come from?**
(read aloud and check <u>all</u> that apply)

- ☐ Declared employment
- ☐ Undeclared or black employment
- ☐ Self-employed
- ☐ Social welfare of last resort- « Revenu d'intégration sociale » (CPAS)
- ☐ Job seekers allowance
- ☐ Child benefits
- ☐ Sickness indemnity
- ☐ Disability indemnity (more than 1-year sick leave)
- ☐ Allowance for disabled person
- ☐ Pension
- ☐ Other **(specify: ____________________________)**

<u>→ For women who are Belgian since birth, → skip to Q101</u>

The following questions concern your administrative situation in Belgium. We are interested in this information because we want to understand the experiences of immigrant women in Belgium in order to better adapt health care. All information provided will remain confidential, and no information will be given to the Immigration Office. Your answers will not affect your immigration application if it is pending.

98. What is your current immigration status?
(Show the card « immigration statuses » if necessary, and check all that apply)

- ☐ Belgian
- ☐ European Union citizen
- ☐ Permanent/unlimited stay
- ☐ Refugee or beneficiary of subsidiary protection
- ☐ Asylum seeker
- ☐ Family reunion
- ☐ Short stay (3 months maximum for family visit, tourism, or medical visit)
- ☐ Stay limited to studies/work
- ☐ Irregular situation/ undocumented migrant
- ☐ Victim of human trafficking
- ☐ Other **(specify :______________________________)**
- ☐ N/A (Don't know) ***(→ skip to Q100)***
- ☐ N/A (Don't want to answer) ***(→ skip to Q100)***

99. How long have you had this status?

- ☐ Since arriving in Belgium
- ☐ Other : ☐ <1 year ☐ 1-5 years ☐ 6-10 years ☐ >10 years ☐ Don't know

100. Are you legally allowed to work in Belgium?

- ☐ Yes
- ☐ No
- ☐ Don't know

The following questions concern your general health

101. Do you have any chronic physical or mental illnesses or conditions? E.g.: diabetes, heart disease, depression, HIV?

- ☐ No
- ☐ Yes **(Specify which: ____)**
- ☐ Don't know

102. **How much did you weigh before you got pregnant?**

__ __ kg / __ __(pounds) __ __(ounces)

☐ Don't know ***(→ see medical notes)***

103. **How much did you weigh before giving birth?**

__ __ kg / __ __(pounds) __ __(ounces)

☐ Don't know ***(→ see medical notes)***

104. **What is your height?**

__ m __ __(cm) / __(feet) __ __ (inches)

☐ Don't know ***(→ see medical notes)***

We have a few questions regarding the planning of this pregnancy.

105. **At the time when you got pregnant with this baby, did you want to get pregnant?**
(read aloud and check one)

☐ Yes
☐ No ***(→Skip to Q107)***
☐ Uncertain ***(→Skip to Q107)***
☐ N/A (doesn't want to answer) ***(→Skip to Q107)***

106. **Was the conception of this baby medically assisted (i.e. using Assisted Reproductive Technologies such as IVF)?**

☐ Yes ***(→Skip to Q110)***
☐ No ***(→Skip to Q110)***
☐ Don't want to answer ***(→Skip to Q110)***

107. **Did you use a contraceptive method to prevent pregnancy, for example condoms, the contraceptive pill, or other methods?**
(For more examples see list in Q108)

☐ Yes
☐ No ***(→Skip to Q109)***
☐ Don't know ***(→Skip to Q109)***

108. What did you use?
(Let the mother answer, then check all answers that apply and skip to Q110)

- ☐ Condom
- ☐ The contraceptive pill
- ☐ Intrauterine device (IUD)
- ☐ Diaphragm/cervical cap
- ☐ Depo-Provera injection
- ☐ Insert under the skin of the arm (Norplant)
- ☐ Breastfeeding
- ☐ Withdrawal ("pull-out")
- ☐ Observation of monthly cycle
- ☐ You thought you or your partner were sterile
- ☐ Abstinence
- ☐ Other **(Specify : ______________________________)**
- ☐ N/A

109. If you did not use something to prevent pregnancy, why not?
(Allow mother to answer and check all that apply)

- ☐ No access to clinic or healthcare provider
- ☐ Side effects
- ☐ Could not afford it
- ☐ Religious reasons
- ☐ Husband/family did not allow it
- ☐ Other (**Specify :** ________________________________)
- ☐ N/A

To conclude, we have some questions regarding you habits and your health

110. In the year preceding your pregnancy, did you smoke any tobacco?

- ☐ No ***(→skip to Q112)***
- ☐ Yes, sometimes
- ☐ Yes, every day or almost every day
- ☐ Yes, but I quit smoking during that year ***(→skip to Q112)***

111. During this pregnancy, did you smoke any tobacco?
(Read aloud and check one)

- ☐ No
- ☐ Yes, sometimes
- ☐ Yes, every day or almost every day
- ☐ Yes, but I quit smoking during my pregnancy

112. During this pregnancy, did you drink any alcohol?
(read aloud and check one)

- ☐ Never
- ☐ Rarely (≤ 1 drink/month)
- ☐ Sometimes (between 2 drinks/month and 1 drink/week)
- ☐ Often (≥ 2 drinks/week)

---

113. <u>At least one month before you became pregnant</u>, did you take prenatal vitamins or folic acid?
(If yes, read aloud the options with "yes" and check one)

- ☐ Yes, almost every day ***(→Skip to Q115)***
- ☐ Yes, from time to time
- ☐ No
- ☐ Don't know ***(→Skip to Q115)***

---

114. If « no » or « from time to time », why?
(Allow the mother to answer and check <u>all</u> that apply)

- ☐ Was not told to take any/ didn't know she was supposed to take any
- ☐ No need
- ☐ Didn't know what they were for
- ☐ Forgot
- ☐ Side effects (e.g. nausea)
- ☐ Couldn't afford them
- ☐ Couldn't find any
- ☐ Hadn't planned the pregnancy
- ☐ Other **(specify : ____)**
- ☐ N/A

---

115. <u>During your pregnancy</u>, did you take prenatal vitamins or folic acid?
(If yes, read aloud the options with "yes" and check one)

- ☐ Yes, almost every day ***(→End of questionnaire)***
- ☐ Yes, from time to time
- ☐ No
- ☐ Don't know ***(→End of questionnaire)***

116. **If « no » or « from time to time », why?**
(Allow the mother to answer and check <u>all</u> that apply)

- ☐ Was not told to take any/ didn't know she was supposed to take any
- ☐ No need
- ☐ Didn't know what they were for
- ☐ Forgot
- ☐ Side effects (e.g. nausea)
- ☐ Couldn't afford them
- ☐ Couldn't find any
- ☐ Other **(Specify : ___________________________)**
- ☐ N/A

This concludes our interview. Thank you very much for your time and for sharing your experience today.
Do you have any questions?
Thank you, I wish you all the best

<u>Definitions:</u>

Miscarriage: foetal death < 24 weeks gestation or <500g

T.O.P.: voluntary termination of pregnancy (in Belgium <12 weeks gestation, other countries <12 or up to 24 weeks)

Medical termination of pregnancy (M.T.O.P.): termination for medical reasons (maternal health or health of the baby) until term

Perinatal and infant mortality: fœtal death > 24 weeks gestation or >500g, death during birth, or in the week following birth. Infant mortality : death in the year following birth.
